# Prediction of PaO_2_ from SpO_2_ values in critically ill invasively ventilated patients: rationale and protocol for a patient–level analysis of ERICC, LUNG SAFE, PRoVENT and PRoVENT–iMiC (PRoPERLy II)

**DOI:** 10.62675/2965-2774.20250270

**Published:** 2025-03-19

**Authors:** Simon Corrado Serafini, David M. P. van Meenen, Luigi Pisani, Ary Serpa, Luciano César Pontes Azevedo, Tai Pham, Eya Sahraoui, Giacomo Bellani, John G. Laffey, Marcus J. Schultz, Guido Mazzinari

**Affiliations:** 1 University of Genoa Department of Surgical Sciences and Integrated Diagnostics Genova Italy Department of Surgical Sciences and Integrated Diagnostics, University of Genoa - Genova, Italy.; 2 Amsterdam UMC Department of Anesthesiology Amsterdam Netherlands Department of Anesthesiology, Amsterdam UMC, location ‘AMC’ - Amsterdam, The Netherlands.; 3 University of Bari "Aldo Moro" Section of Anesthesiology and Intensive Care Medicine Department of Precision-Regenerative Medicine and Jonic Area Bari Italy Department of Precision-Regenerative Medicine and Jonic Area, Section of Anesthesiology and Intensive Care Medicine, University of Bari "Aldo Moro" - Bari, Italy.; 4 Monash University School of Public Health and Preventive Medicine Australian and New Zealand Intensive Care Research Centre Melbourne Australia Australian and New Zealand Intensive Care Research Centre, School of Public Health and Preventive Medicine, Monash University - Melbourne, Australia.; 5 Hospital Israelita Albert Einstein Department of Critical Care Medicine São Paulo SP Brazil Department of Critical Care Medicine, Hospital Israelita Albert Einstein - São Paulo (SP), Brazil.; 6 Université Paris-Saclay Hôpital de Bicêtre Service de Médecine Intensive-Réanimation France Service de Médecine Intensive-Réanimation, Université Paris-Saclay, AP-HP, Hôpital de Bicêtre, DMU CORREVE, FHU SEPSIS, Groupe de Recherche Clinique CARMAS - Le Kremlin - Bicêtre, France.; 7 Université Paris-Saclay Centre de Recherche en Epidémiologie et Santé des Populations Equipe d’Epidémiologie Respiratoire Intégrative Villejuif France Equipe d’Epidémiologie Respiratoire Intégrative, Centre de Recherche en Epidémiologie et Santé des Populations, Université Paris-Saclay, UVSQ, Univ. Paris-Sud, Inserm U1018 - Villejuif, France.; 8 University of Trento Centre for Medical Sciences Trento Italy Centre for Medical Sciences, University of Trento - Trento, Italy.; 9 University of Galway School of Medicine Department of Anaesthesia and Intensive Care Medicine Galway Ireland Department of Anaesthesia and Intensive Care Medicine, School of Medicine, University of Galway - Galway, Ireland.; 10 Amsterdam UMC Department of Intensive Care Amsterdam Netherlands Department of Intensive Care, Amsterdam UMC, location ‘AMC’ - Amsterdam, The Netherlands.; 11 Hospital Universitario la Fe Department of Anesthesiology Valencia Spain Department of Anesthesiology, Hospital Universitario la Fe - Valencia, Spain.

**Keywords:** Critical illness, Critical care, Respiratory distress syndrome, Respiratory insufficiency, Respiration,artificial, Oxygen, Oxygen saturation, Partial pressure, Risk assessment

## Abstract

**Introduction:**

In patients with acute respiratory distress syndrome (ARDS), mortality risk is typically assessed using the arterial partial pressure of oxygen (PaO_2_) divided by the fraction of inspired oxygen (FiO_2_), known as the PaO_2_/FiO_2_ ratio. Recently, the SpO_2_/FiO_2_ ratio, which uses peripheral oxygen saturation (SpO_2_) instead of PaO_2_, has been suggested as a reasonable alternative when there is little access to arterial blood gas analyses. Additionally, equations that predict PaO_2_ from SpO_2_ values could offer another viable method for assessment.

**Aim:**

To evaluate the accuracy of methods for predicting PaO_2_ from SpO_2_ values and compare risk stratification based on measured versus predicted PaO_2_/FiO_2_ ratios using a large database that harmonizes the individual data of patients included in four observational studies.

**Methods and analysis:**

The individual data from four observational studies (‘Epidemiology of Respiratory Insufficiency in Critical Care study’ [ERICC], ‘Large Observational Study to Understand the Global Impact of Severe Acute Respiratory Failure’ [LUNG SAFE], ‘PRactice of VENTilation in critically ill patients without ARDS’ [PRoVENT], and ‘PRactice of VENTilation in critically ill patients in Middle–income Countries’ [PRoVENT–iMiC]) were harmonized and pooled into a database named ‘PRoPERLy II’. The primary endpoint of this planned analysis will be the accuracy of currently available methods to predict PaO_2_ from SpO_2_ values. The secondary endpoint will be the accuracy of classification based on SpO_2_/FiO_2_ ratio and the predicted PaO_2_/FiO_2_ ratio.

**Dissemination:**

Our planned analysis addresses a clinically important research question by comparing different equations for predicting PaO_2_ from SpO_2_ values.

## INTRODUCTION

For patients with acute respiratory distress syndrome (ARDS), mortality risk is commonly stratified by the ratio of the arterial partial pressure of oxygen (PaO_2_) to the fraction of inspired oxygen (FiO_2_), referred to as the PaO_2_/FiO_2_ ratio.^(1)^ In centers where access to repeated arterial sampling is limited or unavailable, such as in resource–limited settings, alternative methods must be considered. Moreover, during pandemic outbreaks with high patient volumes, alternative approaches to repeated sampling could be necessary. This has prompted consideration of SpO_2_/FiO_2_ as an alternative to the PaO_2_/FiO_2_ ratio, where peripheral oxygen saturation (SpO_2_) is a substitute for PaO_2_.^(2)^ However, it remains uncertain whether risk stratification based on the SpO_2_/FiO_2_ ratio performs equivalently to that based on the PaO_2_/FiO_2_ ratio.

Another approach could be to predict PaO_2_ from SpO_2_ values, for which several equations have been suggested.^(3-8)^ However, their use remains limited. Not all equations have been validated in invasively ventilated patients^(7)^ or in adult patients,^(9)^ and inaccuracies may occur, particularly at higher PaO_2_ values.^(3-5,7)^ Newer equations for noninvasive respiratory support patients^(7)^ and critically ill pediatric patients^(9)^ have been suggested, but these equations also need validation in invasively ventilated patients and adult patients.

We have recently established a large database named PRoPERLy II. This database consists of harmonized and pooled individual patient data from four prospective, observational studies named the Epidemiology of Respiratory Insufficiency in Critical Care study (ERICC),^(10)^ the Large Observational Study to Understand the Global Impact of Severe Acute Respiratory Failure (LUNG SAFE),^(11)^ the PRactice of VENTilation in critically ill patients without ARDS (PRoVENT),^(12)^ and the PRactice of VENTilation in critically ill patients in Middle–income Countries (PRoVENT–iMiC).^(13)^ Since these studies collected simultaneously obtained PaO_2_ and SpO_2_ values, along with the corresponding FiO_2_ at the time of collection, this database is well suited for validating various equations for calculating PaO_2_ from SpO_2_ values.

Here, we outline the structure of the PRoPERLy II database and present a detailed analysis plan for its initial analysis, focusing on the accuracy of several suggested methods for predicting PaO_2_. Our primary objective is to assess the accuracy of these equations. The secondary objective is to determine whether classifications based on predicted PaO_2_/FiO_2_ ratios create comparable mortality risk groups to those based on measured PaO_2_/FiO_2_ ratios. We hypothesize that the accuracies of the various equations will differ; however, they will result in similar stratification of patients into severity groups based on PaO_2_/FiO_2_ ratios.

## METHODS

### Study design

**Figure 1:**
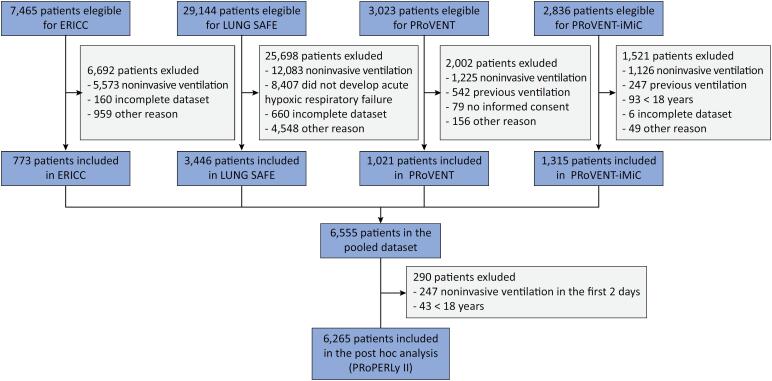
Flow of patients.

PRoPERLy II is a harmonized and pooled database comprising individual data of patients included in four large, prospective, observational studies focusing on ventilation management in critically ill patients, including 773 patients from Brazil in ERICC,^(10)^ 3,446 patients from 50 countries worldwide in LUNG SAFE,^(11)^ 1,021 patients from 16 countries worldwide in PRoVENT,^(12)^ and 1,315 patients from 10 Asian countries in PROVENT–iMiC^(13)^ (Figure 1). PRoPERLy II thus contains data from a total of 6,555 invasively ventilated patients. Further information regarding the original studies can be found in the original publications. The creation of the pooled database, including the harmonization of collected data, required neither additional ethical approval nor individual patient informed consent. Each analysis of PRoPERLy II will follow the Strengthening the Reporting of Observational Studies in Epidemiology (STROBE) Statement.^(14)^

ERICC - Epidemiology of Respiratory Insufficiency in Critical Care; LUNG SAFE - Large observational study to Understand the Global impact of Severe Acute respiratory Failure; PRoVENT - Practice of VENTilation in critically ill patients without ARDS study; PRoVENT–iMiC - Practice of VENTilation in critically ill patients in middle income countries study.

The databases of the 4 original studies were standardized, using case report forms and data dictionaries, and thereafter merged into the pooled database. The original studies met the following inclusion criteria, as reported in table 1.

**Table 1:** Summary table: inclusion and exclusion criteria across trials

Study	Eligibility criteria	Exclusion criteria
ERICC	Aged 18 years or older Admitted to an ICU Receiving invasive mechanical ventilation for a minimum of 24 hours within the initial 48 hours of admission, or NIV for at least 6 hours per day	- Tracheostomy patients - Patients admitted for uncomplicated postoperative care - Readmitted patients - Those with a terminal condition
LUNG SAFE	Admitted to an ICU Receiving invasive ventilation or NIV	- Patients under the age of 16 - Those who did not provide informed consent when deemed necessary
PRoVENT	Aged 18 years or older Admitted to an ICU Receiving invasive ventilation, even if initiated outside the hospital, in the emergency room, clinical ward, or operating room, or if it started in the ICU after admission	- Patients in whom ventilation started before the study recruitment week - Patients receiving only NIV - Those transferred from another hospital under mechanical ventilation
PRoVENT–iMiC	Admitted to an ICU Receiving invasive ventilation, even if initiated outside the hospital, in the emergency room, clinical ward, or operating room	- Patients under 18 years of age - Patients receiving only NIV - Those transferred from another hospital under mechanical ventilation

ERICC - Epidemiology of Respiratory Insufficiency in Critical Care; ICU - intensive care unit; NIV - noninvasive ventilation; LUNG SAFE - Large observational study–Understand the Global impact of Severe Acute respiratory Failure; PRoVENT - Practice of VENTilation in critically ill patients without ARDS study; PRoVENT–iMiC - Practice of VENTilation in critically ill patients in middle-income countries study.

ERICC included patients aged 18 years or older who received ventilatory support for at least 24 hours during the first 48 hours of intensive care unit (ICU) admission at participating ICUs. They excluded patients who underwent tracheostomy, patients admitted for routine uncomplicated postoperative care, patients who were readmitted, and patients with terminal conditions. ERICC enrolled patients over a 2-month period.

LUNG SAFE included patients aged 16 years or older who received invasive or noninvasive ventilation. LUNG SAFE excluded patients who never received invasive ventilation, patients whose electronic case report forms were not fully complete, those who did not develop acute hypoxic respiratory failure, and those without informed consent. LUNG SAFE enrolled patients during a 4-week period during the winter months.

PRoVENT included patients aged 18 years or older who received invasive ventilation. They excluded patients in whom ventilation was started before the study recruitment week, patients receiving only noninvasive ventilation, and patients who were transferred to the ICU from another hospital under mechanical ventilation. PRoVENT enrolled patients during a predefined 1-week period.

PRoVENT–iMiC included patients aged 18 years or older who started with invasive ventilation. They excluded patients who received only noninvasive ventilation, patients whose invasive ventilation started before the inclusion phase of the study, and patients transferred from another hospital while under ventilation. PRoVENT–iMiC enrolled patients during a 4-week period.

All patients included in PRoPERLy II were eligible for participation in an analysis in which we determined the accuracy of various equations for the prediction of PaO_2_ from SpO_2_. For this specific analysis, we will apply additional exclusion criteria: patients without reported SpO_2_ values and those whose SpO_2_ values were recorded more than 2 hours after their corresponding PaO_2_ values. Importantly, these exclusion criteria pertain only to this initial analysis and do not affect the overall dataset creation.

The dataset for PRoPERLy II was constructed to include all eligible patients who received invasive mechanical ventilation, regardless of whether they had a diagnosis of ARDS. Therefore, the complete dataset encompasses both patients with ARDS and those without ARDS, allowing for a comprehensive evaluation of the relationship between SpO_2_ and PaO_2_ values across a diverse patient population. This approach ensures that the analysis is robust and representative of the various underlying conditions leading to acute respiratory failure.

### Data collected in the original studies

In the ERICC, LUNGSAFE, PROVENT, and PROVENT-iMiC studies, the following data were collected. The baseline and demographic variables included age, sex, actual body weight, height, body mass index (BMI), type of admission (medical, elective surgery, urgent surgery, or trauma), and comorbidities, including chronic obstructive pulmonary disease (COPD), diabetes mellitus, chronic kidney disease, neoplasia, neoplasia hematological, heart failure, and chronic liver failure.

The following disease severity score was collected: Sequential Organ Failure Assessment (SOFA). Additionally, for each patient, the main reason for invasive ventilation was reported.

For each patient, ventilation characteristics, including ventilation mode, positive end expiratory pressure (PEEP), tidal volume (V_T_), respiratory rate (RR), peak pressure (Ppeak) or plateau pressure (Pplat), inspired oxygen fraction (FiO_2_), blood gas analysis data when available (arterial pH, partial pressure of arterial blood oxygen tension (PaO_2_), and partial pressure of arterial blood carbon dioxide tension (PaCO_2_)), were collected on Day 1 and Day 2, as captured in the original studies.

### Harmonization and merging

The reasons for invasive ventilation were harmonized and merged into PRoPERLy II, as shown in table 2. These included the predisposing condition according to the lung injury prediction score (LIPS) for several ARDS risk factors. The ventilation days were merged into ‘Day 1’ for the first day of ventilatory variables in patients receiving invasive mechanical ventilation in the ICU and ‘Day 2’ for the second day. Mortality was defined as any death, for all causes, occurring in the ‘ICU or in the hospital (Table 3).

**Table 2:** Data collected across the studies

	Reason for intubation	Data collection frequency
ERICC	- Pneumonia - Neurological - Nonpulmonary sepsis - Asthma/COPD - Cardiogenic pulmonary edema - Extracranial trauma - Hypovolemic/cardiogenic shock - Aspirative syndromes - Others	Data were recorded daily for the first 72 hours after ICU admission
LUNG SAFE	- Medical or surgical admission - Trauma - Neoplastic or immune or hematologic disease - Pneumonia - Pancreatitis - Heart failure	The case report forms automatically prompted investigators to provide an expanded dataset for Days 1, 2, 3, 5, 7, 10, 14, 21, and 28 or at ICU discharge or death. All data were recorded at the same time, normally as close as possible to 10 AM each day. Patient outcomes included date of liberation from mechanical ventilation and vital status at ICU discharge and at either hospital discharge or on Day 90, whichever occurred earlier.
PRoVENT	*Cardiac arrest, Anesthesia for surgery (planned), Depressed consciousness, Respiratory failure*	*Investigators were prompted on the case report form to provide an expanded dataset until Day 7, at discharge from the ICU, and at 90 days after discharge, or after death in the ICU. We counted a ventilation day as any day that the patient received mechanical ventilation, irrespective of the duration of mechanical ventilation for that day and whether or not it was done through an orotracheal tube or tracheostomy.*
PRoVENT-iMiC	*Cardiac arrest, Anesthesia for surgery, Hemodynamic instability, Other, Depressed level of consciousness, Acute respiratory failure (Cause of acute respiratory failure: Community acquired pneumonia, Nosocomial pneumonia, Unplanned postoperative ventilation, Cardiogenic pulmonary edema, Sepsis (other than pneumonia), COPD exacerbation, Other).*	Baseline and demographic variables collected on the day of admission; Extended dataset collected daily until Day 7 and at discharge from the ICU
PRoPERLy II	Patients with ARDS risk factor for: - Aspiration - Pneumonia - Contusion - Trauma - Sepsis - Burns - Pancreatitis - Drug overdose - Unknown	The observations of ventilatory parameters occur on Day 1 and the subsequent evaluations take place the day after in the ICU (labeled as Day 2)

ERICC - Epidemiology of Respiratory Insufficiency in Critical Care; COPD - chronic obstructive pulmonary disease; ICU - intensive care unit; LUNG SAFE - Large observational study–Understand the Global impact of Severe Acute respiratory Failure; PRoVENT - Practice of VENTilation in critically ill patients without ARDS study; PRoVENT–iMiC, Practice of VENTilation in critically ill patients in middle income countries study; ARDS - acute respiratory distress syndrome.

**Table 3:** Outcomes across the studies

	Risk of ARDS or ARDS at admission	Occurrence of ARDS	Length of stay in ICU	Length of stay in hospital	All-cause mortality	Tracheostomy
ERICC	X	X	X	X	X	X
LUNG SAFE	X	X	X	X	X	X
PRoVENT	X	X	X	X	X	X
PRoVENT-iMiC	X	X	X	X	X	X
PRoPERLy II	X	X	X	X	X	X

ARDS - acute respiratory distress syndrome; ICU - intensive care unit; ERICC - Epidemiology of Respiratory Insufficiency in Critical Care; LUNG SAFE - Large observational study–Understand the Global impact of Severe Acute respiratory Failure; PRoVENT - Practice of VENTilation in critically ill patients without ARDS study; PRoVENT–iMiC - Practice of VENTilation in critically ill patients in middle income countries study.

When one study collected a variable differently—for example, by categorizing patients on the Glasgow Coma Scale into groups such as < 7, between 8 and 12, and > 13—the same categorization method was adopted across all studies. During the polling operation, if a variable was not found in at least two datasets and data dictionaries, that variable was excluded.

### Analysis plan

We will assess the accuracy of the following four proposed equations for correctly estimating PaO_2_ and stratifying patient severity based on PaO_2_/FiO_2_ ratio: Rice et al.;^(8)^ Pandariphande et al.;^(6)^ Severinghaus et al.,^(15)^ Ellis ^(3)^ and Gadrey et al.,^(7)^ and Sauthier et al. (Table 4).^(9)^

**Table 4:** Summary table of equations

Pandariphande et al.^(6)^	$\log_{10}\left(\frac{PaO_{2}}{FiO_{2}}\right)=0.48+78\times \log_{10}\left(\frac{SpO_{2}}{FiO_{2}}\right)$
Gadrey et al.^(7)^	$PaO_{2}={\left(\frac{28.6025^{3}}{\frac{1}{SpO_{2}}-0.99}\right)}^{\frac{1}{3}}$
Rice et al.^(8)^	$\frac{PaO_{2}}{FiO_{2}}=\frac{\frac{SpO_{2}}{FiO_{2}}-64}{0.84}$
Sauthier et al.^(9)^	$PaO_{2}={\left(\frac{27.8^{2.8}}{\frac{1}{SpO_{2}}-0.99}\right)}^{\frac{1}{2.8}}$
Severinghaus et al.,^(15)^ Ellis^(3)^	$PaO_{2}={\left(\frac{11700}{SpO_{2}-1}+\sqrt{50^{3}+{\left(\frac{11700}{SpO_{2}-1}\right)}^{2}}\right)}^{\frac{1}{3}}+{\left(\frac{11700}{SpO_{2}-1}+\sqrt{50^{3}+{\left(\frac{11700}{SpO_{2}-1}\right)}^{2}}\right)}^{\frac{1}{3}}$

PaO_2_ - partial pressure of oxygen in arterial blood (mmHg); FiO_2_, - fraction of inspired oxygen; SpO_2_ - peripheral capillary oxygen saturation; log_10_ - logarithm to the base 10.

### Calculations and definitions

V_T_ is expressed in mL/kg predicted body weight (PBW), where PBW is calculated as follows:


[Eq. 1] $$\text{in males, PBW}=50+0.91^{*}(\text{height}(\text{cm})-152.4(\text{cm}))$$

[Eq. 2] $$\text{in females, PBW}=45.5+0.91^{*}(\text{height}(\text{cm})-152.4(\text{cm}))$$

For RR, expressed in breath/min, we use the total RR as reported in the original studies.

The dynamic driving pressure (ΔP) is expressed in cm H_2_O and is calculated as follows:


[Eq. 3] $$\Delta \text{P}=\text{Pplat}\left({\text{cmH}}_{2}\text{O}\right)-\text{PEEP}\left({\text{cmH}}_{2}\text{O}\right)(\text{in volume-controlled ventilation})$$

[Eq. 4] $$\Delta \text{P}=\text{Ppeak}\left({\text{cmH}}_{2}\text{O}\right)-\mathrm{PEEP}\left({\text{cmH}}_{2}\text{O}\right)(\text{in pressure-controlled ventilation})$$

The dynamic compliance respiratory system (C_RS_), expressed in ml/cm H_2_O, is calculated as follows:


[Eq. 5] $${\text{C}}_{\text{RS}}={\text{V}}_{\text{T}}(\text{ml})/\Delta \text{P}\left({\text{cmH}}_{2}\text{O}\right)$$

The dynamic mechanical power (MP) is expressed in J/min and is calculated as follows:


[Eq. 6] $$\text{MP}=0.098*{\text{V}}_{\text{T}}*\text{RR}*(\mathrm{Pmax}-0.5*\Delta \text{P})$$

### Endpoints

The primary endpoint is the accuracy of currently available methods to predict PaO_2_ from SpO_2_ values. The secondary endpoint is the accuracy of classification based on SpO_2_/FiO_2_ ratio and the predicted PaO_2_/FiO_2_ ratio.

### Sample size

No formal sample size calculation was performed. The sample size for this initial analysis of PRoPERLy II was based on the number of available patients in the pooled database. In anticipation of a 60% dropout rate due to the application of exclusion criteria for this analysis, we expect to include at least 2,500 patients. The number of missing data points will be reported in a specific table in the supplement, and no imputation will be used.

### Statistical analysis plan

We will use simultaneously obtained PaO_2_ and SpO_2_ values, along with the corresponding FiO_2_, if the timeframe does not exceed 2 hours. We will fit each estimation method to the data to obtain a predicted PaO_2_ and predicted PaO_2_/FiO_2_ ratios using these estimated values. All the models will be validated with 500 bootstrap repetitions to improve the robustness of the effect estimates and control for overfitting. PaO_2_ will be measured via arterial blood gas (ABG) analysis. The accuracy of the predicted PaO_2_ will be assessed via the root mean squared error (RMSE), mean absolute error (MAE) and mean absolute percentage of error (MAPE) of the derived models. ARDS severity will be classified according to the Berlin criteria cutoff (ordinal score) by Somers´ Dxy rank correlation. The accuracy of the predictions will be further evaluated with the Diebold–Mariano t–test. Moreover, calibration plots and intraclass correlation coefficient will be reports as recommended.^(16)^

Additionally, precision error, bias, and limits of agreement will be assessed with Bland–Altman analysis, where the reference method is the measured PaO_2_. The threshold of acceptable bias and precision (defined as the difference between the upper and lower LoAs) was preestablished at 19 to account for the variability of ABG measurements.^(17)^ The precision error will be calculated as follows: 1.96 × standard deviation (SD) of the bias of the methods × 100%.^(18)^ The normality distribution of the differences will be checked with quantile–quantile (QQ) plots and histograms. If the normality assumption is not fulfilled, logarithmic data transformation will be performed. Proportional bias will be assessed by fitting an ordinary linear regression in Bland–Altman plots.^(19)^ If repeated measures are obtained from patients, confidence intervals for LoA will be adjusted as recommended.^(20)^ Trending ability will be assessed in addition to accuracy analysis. We use the four-quadrant plot, polar plot, and clinical concordance plot methods to test the magnitude and directionality of the change.^(21)^

The amount of fixed and proportional bias will also be assessed with orthogonal (Deming) regression. Orthogonal regression is a variant of least products regression analysis and allows for both the y and the x values to be attended by random errors. It depends on minimizing the sum of the products of the deviations of both the x and y values from the estimated regression line. Orthogonal regression requires no judgment on whether the y or x variables provide ‘true’ or ‘benchmark’ values. If the scatter increases with the level of y (and x), then weighted least products (WLP) Passing–Bablok regression analysis will be used.^(22)^

After obtaining the best method of predicting PaO_2_ among those tested, we will plot a table of predicted PaO_2_ values according to the administered FiO_2_ and measured SpO_2_ to provide a rapid and easy assessment method.

The accuracy of each estimation in stratifying ARDS severity as defined by the Berlin criteria^(1)^ with respect to the measured PaO_2_/FiO_2_ ratio by Somers’ Dxy rank correlation, a generalization of the receiver operating characteristic area for ordinal variables. Dxy will be calculated after correction for overfitting by cross-validation.

We will perform a sensitivity analysis of the accuracy by analyzing only values below an SpO_2_ of 98%. As a secondary sensitivity analysis, we will reanalyze the data with SpO_2_ values ≤ 96% excluded, recalculating correlations with PaO_2_ to examine the effect of removing these higher saturation levels. In addition, we will perform the following subgroup analysis to assess the effects of the following parameters on the accuracy of the different methods:

1. pH, i.e., < 7.35, 7.35 to 7.45, or > 7.45;
2. PEEP, i.e., < 10 cmH_2_O and high PEEP, i.e., > 10cmH_2_O

## DISCUSSION

This will be the first analysis of PRoPERLy II, a pooled database that harmonizes and merges individual patient data from critically ill patients included in four previously conducted global studies of invasive ventilation. The primary objective of this initial analysis was to assess the accuracy of the currently available methods for predicting PaO_2_ from SpO_2_ values. The secondary objective of this study was to assess the accuracy of the classification of ARDS severity by using predicted PaO_2_/FiO_2_ ratios compared with measured PaO_2_/FiO_2_ ratios in various patient categories.

This analysis addresses a clinically important research question by comparing different equations for predicting PaO_2_ from SpO_2_ values. This investigation is crucial, as accurate prediction of PaO_2_ from SpO_2_ can inform clinical decisions, such as oxygen therapy management and even respiratory support strategies. By evaluating various prediction equations, this study aims to identify the most reliable method for noninvasively estimating arterial oxygenation status, particularly in settings where direct arterial blood gas measurements may be limited or impractical.

The initial analysis of PRoPERLy II may confirm whether the SpO_2_/FiO_2_ ratios constitute a reasonable alternative in settings with little access to arterial blood gas analyses, potentially aiding inclusion in observational studies, facilitating risk stratification, and enabling timely enrollment in randomized clinical trials.

This study is based on data from the PRoPERLy II dataset, which has certain limitations. The four parent studies were conducted independently and in different years, potentially introducing variability in data collection methods, patient populations, and clinical practices. Additionally, we lacked access to variables such as shock status (i.e., use of vasopressors), skin color, and distal perfusion quality, as these variables were not collected in the original studies; this limits our ability to assess certain factors that may influence SpO_2_ measurement accuracy. This specific analysis has additional limitations. Indeed, while previous findings show^(7)^ acceptable SpO_2_ and PaO_2_ pairing with measures taken up to 30 minutes apart, reducing the timeframe to under 30 minutes was not feasible with the data available from the original studies. As a post hoc analysis of previously collected observational data, the findings can be interpreted only as hypothesis–generating and need to be confirmed through prospective studies to establish causality. Despite these limitations, this analysis provides valuable insights that could guide future research and clinical practice. The application of robust statistical methods, including multivariable models, ensures the reliability and validity of the results. By strictly adhering to a predefined statistical analysis plan, the study minimizes the risk of deviations from the initial research hypotheses, thereby maintaining the integrity and credibility of the analysis. The strengths of the analysis derive partially from the extensive PRoPERLy II database, which enables sophisticated and comprehensive analyses, enhancing the statistical power and reliability of the findings. Additionally, the inclusion of global participation, encompassing data from various types of hospitals in both resource-rich and resource-limited settings, increases the generalizability of the results.

Notably, after this initial analysis of PRoPERLy II, the database will be available for additional analysis. For this purpose, interested investigators will have to provide a detailed analysis plan, containing a testable hypothesis, clearly described endpoints and an analysis plan. After approval, investigators will be provided with a mock database that will allow them to write an analysis script in R. PROVE network investigators will then run the script on the PRoPERLy II database and provide the investigators with the results. Any publication from an additional analysis must follow the rules for publication denoted by the PROVE network.^(23)^
