# Erratum: Prediction of PaO_2_ from SpO_2_ values in critically ill invasively ventilated patients: rationale and protocol for a patient–level analysis of ERICC, LUNG SAFE, PRoVENT and PRoVENT–iMiC (PRoPERLy II)

**DOI:** 10.62675/2965-2774.20250270err

**Published:** 2025-09-09

In the article **Prediction of PaO**_2_ **from SpO**_2_ **values in critically ill invasively ventilated patients: rationale and protocol for a patient–level analysis of ERICC, LUNG SAFE, PRoVENT and PRoVENT–iMiC (PRoPERLy II)**, with DOI number: 10.62675/2965-2774.20250270, published in the journal Critical Care Science, 2025;37:e20250270, on page 6, table 4, fifth line:

**Where it read:**

**Table:** 

Severinghaus et al., ^(15)^ llis^(3)^	$PaO_{2}={\left(\frac{11700}{SpO_{2}-1}+\sqrt{50^{3}+{\left(\frac{11700}{SpO_{2}-1}\right)}^{2}}\right)}^{\frac{1}{3}}+{\left(\frac{11700}{SpO_{2}-1}+\sqrt{50^{3}+{\left(\frac{11700}{SpO_{2}-1}\right)}^{2}}\right)}^{\frac{1}{3}}$

**Read:**

**Table:** 

Severinghaus et al.,^(15)^ Ellis^(3)^	$PaO_{2}={\left(\frac{11700}{SpO_{2}-1}+\sqrt{50^{3}+{\left(\frac{11700}{SpO_{2}-1}\right)}^{2}}\right)}^{\frac{1}{3}}+{\left(\frac{11700}{SpO_{2}-1}+\sqrt{50^{3}-{\left(\frac{11700}{SpO_{2}-1}\right)}^{2}}\right)}^{\frac{1}{3}}$

